# Reduced frequency of lateral root branching improves N capture from low-N soils in maize

**DOI:** 10.1093/jxb/erv007

**Published:** 2015-02-13

**Authors:** Ai Zhan, Jonathan P. Lynch

**Affiliations:** ^1^State Key Laboratory of Soil Erosion and Dryland Farming on the Loess Plateau, Northwest A&F University, Yangling, Shaanxi 712100, China; ^2^Department of Plant Science, The Pennsylvania State University, University Park, PA 16802,USA

**Keywords:** Branching, frequency, lateral root, maize (*Zea mays*), nitrogen (N), respiration.

## Abstract

Suboptimal nitrogen (N) availability is a primary constraint for crop production in developing countries, while in developed countries, intensive N fertilization is a primary economic, energy, and environmental cost for crop production. We tested the hypothesis that under low-N conditions, maize (*Zea mays*) lines with few but long (FL) lateral roots would have greater axial root elongation, deeper rooting, and greater N acquisition than lines with many but short (MS) lateral roots. Maize recombinant inbred lines contrasting in lateral root number and length were grown with adequate and suboptimal N in greenhouse mesocosms and in the field in the USA and South Africa (SA). In low-N mesocosms, the FL phenotype had substantially reduced root respiration and greater rooting depth than the MS phenotype. In low-N fields in the USA and SA, the FL phenotype had greater rooting depth, shoot N content, leaf photosynthesis, and shoot biomass than the MS phenotype. The FL phenotype yielded 31.5% more than the MS phenotype under low N in the USA. Our results are consistent with the hypothesis that sparse but long lateral roots improve N capture from low-N soils. These results with maize probably pertain to other species. The FL lateral root phenotype merits consideration as a selection target for greater crop N efficiency.

## Highlight

Maize genotypes producing fewer lateral roots extend them further into the soil, improving overall plant N acquisition.

## Introduction

Suboptimal nitrogen (N) availability is a primary limitation to plant growth in terrestrial ecosystems (Tilman *et al.*, 2002). In poor countries, low-N availability is a principal, pervasive constraint to crop production and therefore food security and economic development, as most smallholder farmers have limited access to fertilizer (Azeez *et al.*, 2006; Worku *et al.*, 2007). In developed countries, intensive N fertilization sustains high yields, but N use is generally inefficient, with only 30–40% of total N applied actually being harvested in grain (Raun and Johnson, 1999). Much of the remaining applied N is lost as surface runoff, leached nitrate (NO_3_^–^) (London, 2005), or gaseous losses (Beman *et al.*, 2005), all of which pose environmental concerns (Stulen *et al.*, 1998). It is estimated that a 1% increase in N utilization efficiency could save ~$1.1 billion annually (Kant *et al.*, 2011). Therefore, improved N efficiency would afford multiple global benefits.

The majority of research to improve N efficiency has focused on shoot biomass and grain yield as selection criteria (e.g. Moll *et al.*, 1982; Raun and Johnson, 1999; Raun *et al.*, 2002), and opportunities in the longer term to improve N transport and assimilation (e.g. Andrews *et al.*, 2004; Hirel *et al.*, 2007; Shrawat *et al.*, 2008; Kant *et al.*, 2011). The relevance of root traits for efficient N capture has received less attention. Root architecture has key importance for nutrient and water acquisition by positioning root foraging in specific soil domains in time and space (Lynch, 1995, 2011). For example, the ‘topsoil foraging’ ideotype appears to be particularly important for genotypic adaptation to low-phosphorus (P) soils (Lynch, 2011; Lynch and Brown, 2001; Richardson *et al.*, 2011). Nitrate, the dominant form of N in most agricultural soils, is highly soluble and is therefore subject to leaching into deeper soil strata (Thorup-Kristensen *et al.*, 2009). Root systems with rapid exploitation of deep soil would optimize N capture in most production environments (Lynch, 2013). The ‘Steep, cheap, and deep’ (SCD) root ideotype consists of architectural, anatomical, morphological, and physiological phenes that work together to improve the capture of water and N in leaching environments by accelerating subsoil exploration (Lynch, 2013). Our focus here is on lateral root branching and length. According to the SCD ideotype, the frequency and length of lateral roots is important for N capture (Lynch, 2013). In the context of a cost/benefit analysis (Zhu and Lynch, 2004; Lynch and Ho, 2005; Zhu *et al.*, 2005), N would be captured more efficiently by phenotypes with fewer but longer laterals capable of exploring a greater volume of soil accessible via mass flow of water, and therefore nitrate, than would phenotypes with a greater number of short laterals of equivalent total length. On the other hand, sparse lateral branching should concentrate internal resources on axial elongation and thereby increase rooting depth, and should reduce competition for N among neighbouring lateral roots. This prediction is supported by results from the functional-structural plant model *SimRoot* (Postma *et al.*, 2014). Results from *SimRoot* indicate that sparse, long laterals were are optimal for N acquisition by decreasing competition among lateral roots for nitrate and reducing metabolic costs for root construction and maintenance. By reducing competition among lateral roots, sparse, long laterals effectively increase N uptake per unit root length, and by decreasing the metabolic cost, sparse, long laterals permit internal reallocation of nutrients to axial elongation, which is particularly beneficial under conditions of low-N availability (Postma and Lynch, 2011).

Lateral roots emerge from axial roots from lateral root primordial that initiate from pericycle founder cells (Malamy and Benfey, 1997; De Smet, 2007; Nibau *et al.*, 2008; Péret *et al.*, 2009).The formation of lateral roots is a major determinant of root system architecture (Nibau *et al.*, 2008; Pérez-Torres *et al.*, 2008). Genotypic differences in lateral root number and length have been reported in different species (Fitter, 1996; Fitter *et al.*, 2002; Zhu *et al.*, 2005; Trachsel *et al.*, 2011; Lynch, 2013; Postma *et al.*, 2014). In maize, Trachsel *et al.* (2011) observed significant genotypic variation in the density of lateral root branching, ranging from no lateral roots to 20 roots cm^–1^. Also, in many *Liliaceae* and *Orchidaceae*, root systems of plants vary greatly in their architecture, from unbranched to highly complex branching patterns (Fitter, 1996; Fitter *et al.*, 2002). Although lateral branching is a heritable trait (Zhu *et al.*, 2005) and genes affecting lateral branching have been identified in several species, including maize (Doebley *et al.*, 1995) and rice (Takeda *et al.*, 2003), several studies report that lateral root number and length can be modulated by external NO_3_^–^ availability (Drew and Saker, 1975; Robinson, 1994; Zhang and Forde, 1998; Linkohr *et al.*, 2002; Hodge, 2004). For instance, in barley, external NO_3_^–^ supply increased numbers of lateral roots and increased rates of lateral root elongation (Drew and Saker, 1975). It is reported that the stimulation of lateral root elongation appears to be attributable to a signalling effect from the NO_3_^–^ ion itself rather than to a downstream metabolite (Zhang and Forde, 1998; Zhang *et al.*, 1999). In *Arabidopsis*, the localized stimulatory effect of external nitrite on lateral root growth depends on the expression of the MADS-box transcription factor ANR1, which acts downstream of the dual-affinity nitrate transporter NRT1.1; both of them act together as an NO_3_^–^ sensor, promoting auxin transport (Zhang and Forde, 1998; Remans *et al.*, 2006; Krouk *et al.*, 2010; Gojon *et al.*, 2011).

The formation of lateral roots increases the sink strength of the root system, promoting the development of greater root length and thereby greater nutrient and water acquisition (Postma *et al.*, 2014). However, in *Medicago truncatula*, the highly branched root architecture genotype TR185 showed a permanently N-starved phenotype (Bourion *et al.*, 2014). Results from the functional-structural plant model *SimRoot* indicate that the optimal lateral root-branching density for N capture is less than that for P capture in maize (Postma *et al.*, 2014). This is mainly because P is poorly mobile and well buffered in soil: most P is acquired within 1mm of the root surface. If root spacing is, on average, >2mm, some soil will remain unexploited. In contrast, nitrate diffusion in soil is three or four orders of magnitude faster than phosphate diffusion, so roots 10mm apart will probably compete for NO_3_^–^ after *~*1 day (Nye and Tinker, 1977). Therefore, the overlap of N-depletion zones around roots of the same plant effectively reduces nitrate uptake efficiency (Berntson, 1994; Fitter *et al.*, 2002). In addition, following the economic paradigm of plant resource allocation (Bloom *et al.*, 1985), root construction and maintenance requires metabolic investment, which can exceed 50% of daily photosynthesis (Lambers *et al.*, 2002). Production of more lateral roots than are needed for N capture would divert carbon and other resources from other root classes, including axial roots (Borch *et al.*, 1999; Miller *et al.*, 2003; Lynch, 2007), potentially slowing axial root elongation into deep soil strata. This is especially important for the acquisition of mobile resources which can accumulate in deep soil strata, like water and nitrate (Lynch, 2013). The few/long (FL) lateral root phenotype is therefore an element of the SCD ideotype for efficient N capture because sparse lateral branching should conserve internal resources, reduce competition for N among neighbouring lateral roots, and explore a greater volume of soil than a many/short (MS) lateral root phenotype.

The overall objective of this research was to assess the utility of lateral root number and length for N acquisition in maize under N-limiting conditions. Specifically, we tested the hypothesis that reduced lateral root number and increased lateral root length are associated with decreased root respiration, greater rooting depth, enhanced N acquisition, and greater plant growth and yield under N limitation.

## Materials and methods

### Greenhouse mesocosm study

#### Plant materials, experimental design, and growth conditions 

Eighteen recombinant inbred lines (RILs) of maize (*Zea mays* L.) from the intermated B73 × Mo17 population (IBM; Supplementary Table S1) were obtained from Shawn Kaeppler (University of Wisconsin, Madison, WI, USA), originally supplied by Charles Stuber and Lynn Senior at North Carolina State University (Senior *et al.*, 1996; Kaeppler *et al.*, 2000). The 18 RILs with contrasting lateral root-branching density and length were chosen according to previous experiments (Trachsel *et al.*, 2011, 2013): nine RILs with FL lateral roots and nine with MS lateral roots. The greenhouse experiment was a randomized complete block design with a 2×18 factorial arrangement of treatments. The factors were two N levels: optimum N (high N, 4.5mM) and suboptimal N (low N, 0.3mM), and 18 genotypes. Four replicates were staggered seven days between replicates with time of planting treated as a block effect.

Seeds of 18 genotypes were surface sterilized in 0.05% NaOCl for 15min and imbibed for 24h in aerated 1mM CaSO_4_, then placed in darkness at 28±1°C for 2 days. Seedlings of similar size were transplanted to mesocosms consisting of polyvinylchloride (PVC) cylinders 15.7cm in diameter and 155cm in height. The cylinders were lined inside with plastic sleeves made of 4 mil (0.116mm) transparent hi-density polyethylene film, which were used to facilitate root sampling. The growth medium consisted of a mixture (volume based) of 50% medium size (0.5–0.3mm) commercial grade sand (Quikrete Companies Inc., Harrisburg, PA, USA), 40% horticultural size #3 vermiculite, and 10% perlite (Whittemore Companies Inc., Lawrence, MA, USA). Twenty-nine litres of the mixture were used in each cylinder. Two days before planting, the cylinders were irrigated with 4.7 L of a nutrient solution adjusted to pH 6.0 and consisting of (in μM): NO_3_ (4500), NH_4_ (300), P (500), K (1000), Ca (1750), SO_4_ (1500), Mg (1000), B (46), Mn (9), Zn (7), Cu (0.32), Mo (0.80), and EDTA-Fe (77). For the low-N treatment, NO_3_ and NH_4_ were reduced to 300 and 20 μM, respectively, and K_2_SO_4_ was used to replace K and SO_4_. Each cylinder received three plants; after 7 days they were thinned to one plant. The plants were grown in a temperature-controlled greenhouse in University Park, PA, USA (GH) (40°48′N, 77°51′W) with a photoperiod of 14/10h at 28/24°C (light/darkness). Following seedling establishment, 200ml of nutrient solution with N was applied in the high-N treatment every 2 days via drip irrigation using a DI-16 Dosatron fertilizer injector (Dosatron International Inc, Dallas, TX, USA). In the low-N treatment, 200ml of nutrient solution without N was supplied every 2 days.

#### Chlorophyll content and net photosynthesis rate 

Plants were harvested 6 weeks after transplanting. Two days before harvest, leaf chlorophyll content (SPAD) and net photosynthesis rate (Pn) were measured. The SPAD readings were measured by using a chlorophyll meter (SPAD 502, Konika Minolta Sensing Inc., Osaka, Japan). For each plant, the SPAD reading was measured in upper, middle, and lower portions of the third youngest fully expanded leaf, and the average of all values for each plant is presented. Leaf gas exchange of the third youngest fully expanded leaf was measured with a Licor-6400 Infrared Gas Analyser (Li-Cor Biosciences, Lincoln, NE, USA) using a red-blue light at PAR intensity of 1200 μmol photons m^–2^ s^–1^, constant CO_2_ concentration of 400 ppm, and leaf temperature of 25°C. The relative humidity was 40%.

#### Root respiration 

Root respiration of axial and lateral roots was measured. Three 10cm root segments from the third whorl of crown roots were excised 15cm from the base. Lateral roots of axial roots were removed with a Teflon blade (Electron Microscopy Sciences, Hatfield, PA, USA). Excised axial and lateral root samples were patted dry and placed in a 40ml custom chamber connected to the Li-6400 IRGA (LI-COR, Lincoln, NE, USA) separately. The temperature of the chamber was maintained at 25±1°C using a water bath while respiration was measured. The CO_2_ evolution from root segments was recorded every 5 s for 180 s (Zhu *et al.*, 2010; Chimungu *et al*, 2014).

#### Shoot and root sampling and analysis 

The shoot was severed at the soil surface, oven-dried at 70°C for 72h, and shoots were ground for total-N analysis after biomass determination. Roots were separated from the soil by vigorously rinsing at low pressure with water. Roots were extracted from each 20cm soil depth increment, three 5cm root segments were taken from each whorl of crown root, primary root, and seminal root (8cm from the top of every soil depth), and lateral root length and lateral root number were obtained by scanning with image analysis software (WinRhizo Pro, Régent Instruments, Québec, Canada). Three roots were randomly chosen from each whorl of crown roots and seminal roots to determine axial root length. Total root length was obtained by scanning with image analysis software (WinRhizo Pro, Régent Instruments, Québec, Québec City, Canada). The N concentration was determined by using an elemental analyser (PerkinElmer SeriesII CHNS/O Analyzer 2400, Shelton, CT, USA).

### Field study

#### Experimental site 

Experiments were carried out during May to August in 2013 at the Russell E. Larson Research and Education Center of the Pennsylvania State University in Rock Springs, PA, USA (RS) (40°42′37″.52 N, 77°57′07″.54W, 366 masl), and during the November in 2013 to January in 2014 season in Alma, Limpopo, Republic of South Africa (24°33′00.12 S, 28°07′25.84 E, 1235 masl). The soils at the experimental sites were a Hagerstown silt loam (fine, mixed, semiactive, mesic Typic Hapludalf) in RS and a Clovelly loamy sand (Typic Ustipsamment) in SA.

#### Plant materials, experimental design, and field conditions 

Based on soil analysis at the beginning of the cropping season, all high-N plots in RS were fertilized with 150kg N ha^−1^ as urea. The plots assigned to the N-deficiency treatment did not receive any N fertilizer. In SA, high-N plots received 207kg N ha^−1^ urea, and low-N plots received 33kg ha^−1^ urea. In both locations, other nutrients were adjusted to meet the requirements for maize production as determined by soil tests. Pest control and irrigation were carried out as needed. Seeds of ten IBM RILs were planted on 17 May 2013 in RS and 28 November 2013 in SA. The experiments were arranged in a split-plot design replicated four times with high- and low-N treatments as the whole plot factor, and genotype as the split plot. Five-row plots of each genotype were randomly assigned within each whole plot. Each row was 4.5 m long, 75cm wide, and distance within a row was 23cm, resulting in a planting density of 6 plants m^–2^.

#### Sampling and analysis 

Shoots and roots were harvested 14 weeks after planting in RS and 13 weeks after planting in SA. Two days before harvest, SPAD and Pn were recorded in the ear leaf (at SA, only SPAD was taken on the third youngest leaf). SPAD and Pn were measured as described above except PAR intensity was set to 1800 μmol photons m^–2^ s^–1^, with a constant CO_2_ concentration of 400 ppm and leaf temperature of 25°C. The relative humidity was 40%. Three adjacent plants were randomly selected in the same row for shoot dry weight per replicate measurement, and oven dried at 70°C for 72h before being weighed. The shoots were ground and 2–3mg ground tissues were used for tissue-N analysis as described above. Roots were excavated by removing a soil cylinder *~*40cm diameter and 25cm depth with the plant base as the horizontal centre of the soil cylinder. The excavated root crowns were cleaned by vigorous rinsing at low pressure with water. The clean roots were subsequently used to measure lateral root number. Three 5cm root segments were taken 5cm from the base of each whorl of crown root, primary root, and seminal root, and lateral root number of corresponding nodal roots was based on counts. All roots emerging belowground were classified as crown roots.

Soil cores of 5.1cm diameter and 60cm length (Giddings Machine Co., Windsor, CO, USA) were taken within a planting row midway between two plants. Each soil core was subdivided into 10cm segments and roots were extracted from each segment. Subsequently extracted root samples were scanned with image analysis software (WinRhizo Pro, Régent Instruments, Québec, Canada) to obtain the root length in each soil depth. Percentages of root length at each depth were calculated in each soil core. Depth above which 95% (D_95_) of root length is located was calculated by linear interpolation between the cumulative root lengths as described in Trachsel *et al.* (2013).

### Data analysis

The experimental data were statistically analysed by one-way ANOVA with R version 3.0.2 (R Development Core Team, 2014). Two-way ANOVA was used for comparisons between FL and MS lines, N levels, and the interaction between these main effects. Probabilities of significance were used to test differences among treatments, and Tukey’s Honest Significant Difference method (*a* = 0.05) was used to compare the means.

## Results

### Lateral root branching and length

**Fig. 1.**
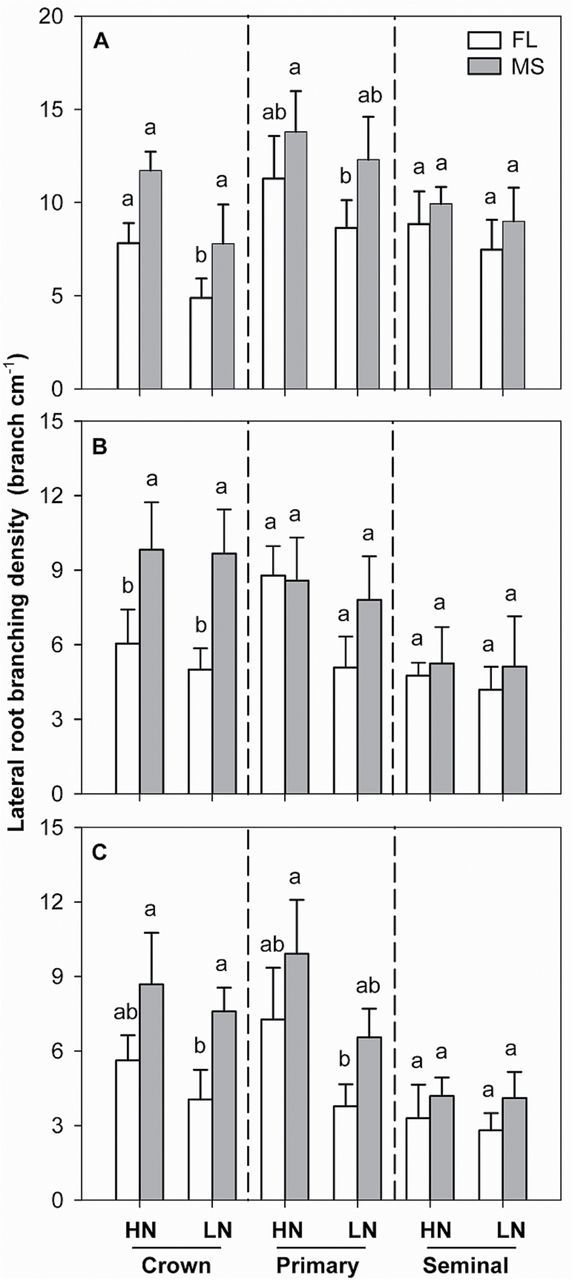
Lateral root-branching density of crown, primary, and seminal roots under high-N (HN) and low-N (LN) conditions at 42 days after planting in GH mesocosms (A), at 14 and 13 weeks after planting in the field in RS (B) and SA (C). The data shown in GH mesocosms are means of four replicates of the nine genotypes, and in RS and SA are means of four replicates of the five genotypes in each phenotypic class in either HN or LN ± SE. Different letters represent significant differences (*P* < 0.05) compared within each root class.

**Fig. 2.**
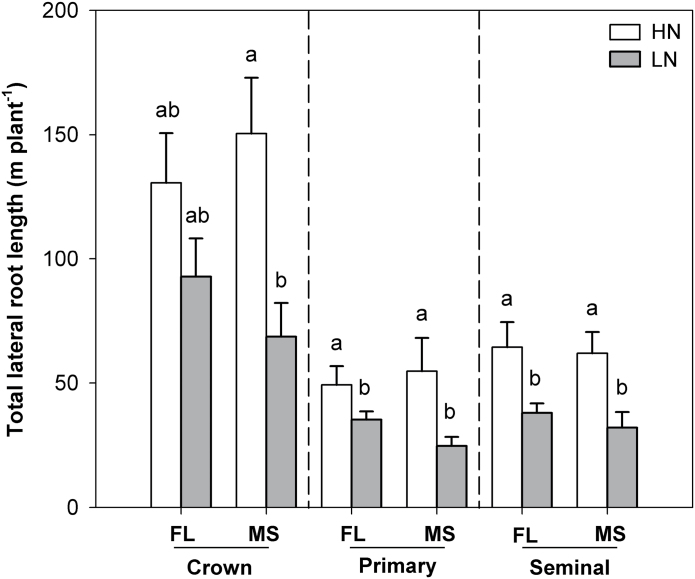
Total lateral root length of crown, primary, and seminal roots under high-N (HN) and low-N (LN) conditions at 42 days after planting in GH mesocosms. The data shown are means of four replicates of the nine genotypes in each phenotypic class in either HN or LN ± SE. Different letters represent significant differences (*P* < 0.05) compared within each root class.

In mesocosms, the lines displayed the expected phenotypes, with MS lines having greater lateral root-branching density of crown roots than FL lines, although no significant difference was found in seminal and primary roots (Fig. 1A). Low N did not significantly decrease lateral root-branching density in either FL or MS lines. In the field experiments, MS lines had greater lateral root-branching density of crown roots than FL lines (Fig. 1B, C). Lateral root-branching density of crown, primary, and seminal roots was not influenced by N treatment in either RS or SA. In the mesocosms, under high-N conditions, the FL and MS lines had equivalent total lateral root length. Low N significantly decreased total lateral root length of all root classes (Fig. 2).

### Effects of lateral root-branching density on root growth

**Fig. 3.**
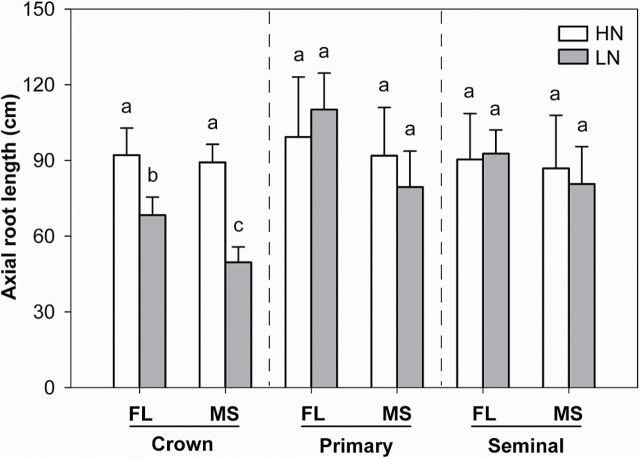
Axial root length of crown, primary, and seminal roots under high-N (HN) and low-N (LN) conditions at 42 days after planting in greenhouse mesocosms. The data shown are means of four replicates of the nine genotypes in each phenotypic class in either HN or LN ± SE. Different letters represent significant differences (*P* < 0.05) compared within each root class.

**Fig. 4.**
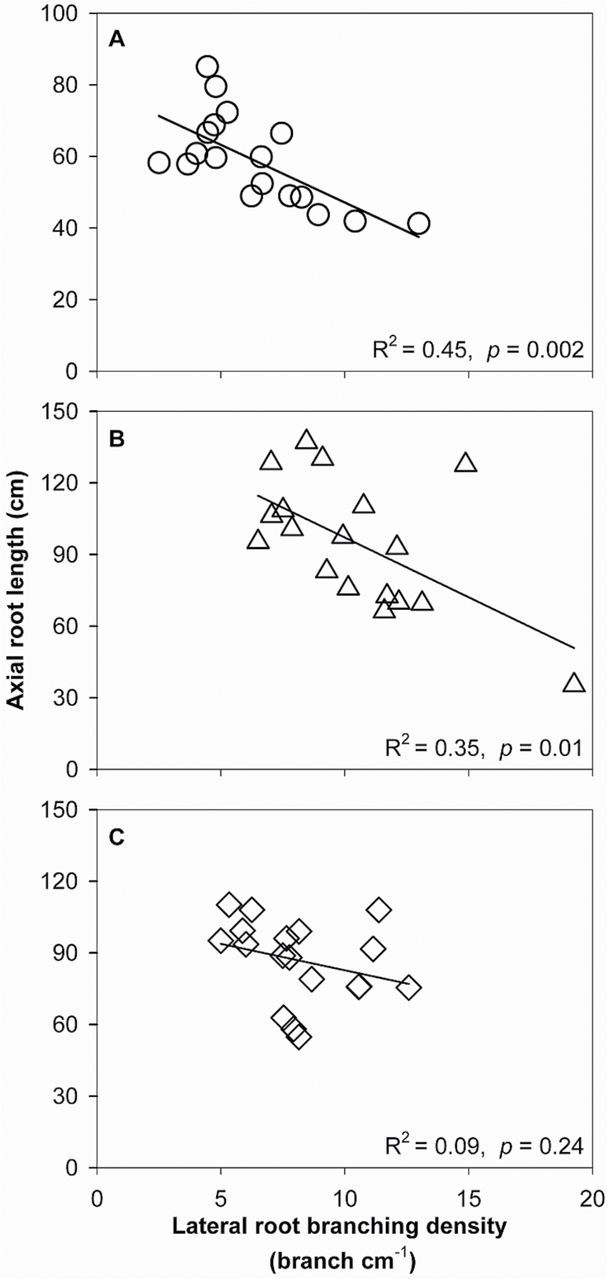
Correlation of axial root length with lateral root-branching density of crown roots (A), primary roots (B), and seminal roots (C) in GH mesocosms at 42 days after planting. Each point is the mean of four replicates of each genotype.

**Fig. 5.**
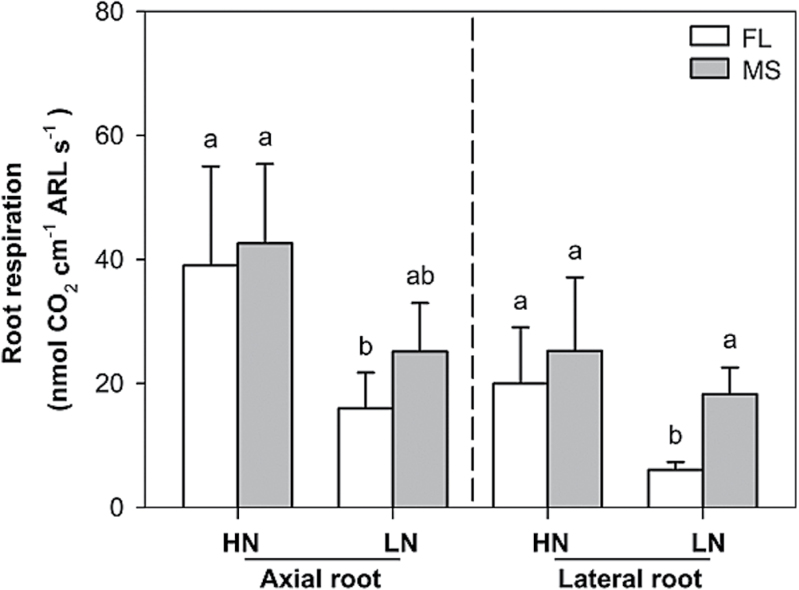
Root respiration of axial and lateral roots per unit ARL of FL and MS lines under high-N (HN) and low-N (LN) conditions at 42 days after planting in GH mesocosms. The data shown are means of four replicates of the nine genotypes in each phenotypic class in either HN or LN ± SE. Different letters represent significant differences (*P* < 0.05) compared within each root class.

**Fig. 6.**
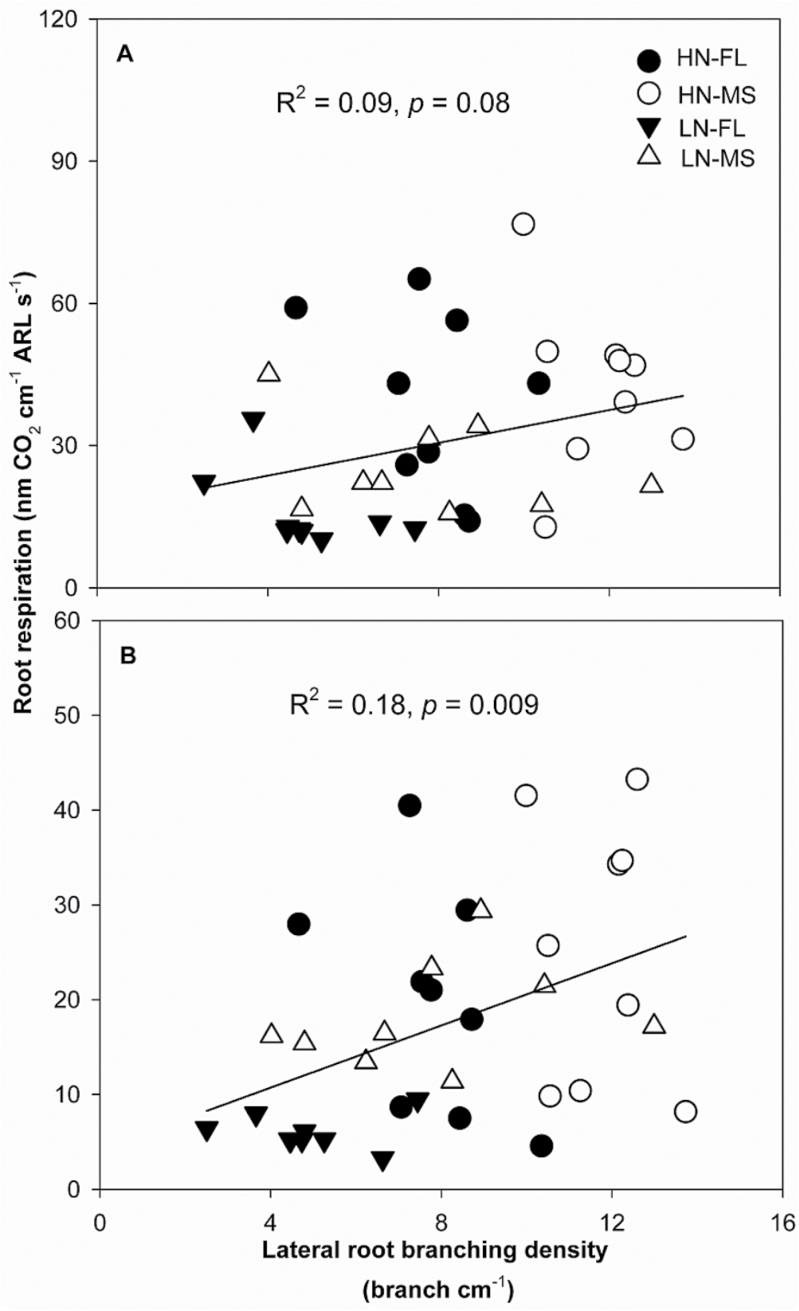
Correlation of axial root respiration per unit ARL (A), and lateral root respiration per unit ARL (B), with crown root lateral root-branching density as affected by high N (circles) and low N (triangles) at 42 days after planting for nine FL (filled symbols) and nine MS (open symbols) genotypes. Each point is the means of four replicates of each genotype.

Under low N in mesocosms, axial root length (ARL) of crown roots was significantly different between FL and MS lines, though no difference was found in primary and seminal roots (Fig. 3). In low-N conditions, crown, primary, and seminal roots of FL lines had ~38, 39, and 15% greater ARL, respectively, than MS lines. The ARL of crown and primary roots was negatively correlated with lateral root-branching density (Fig. 4A, B). N limitation reduced root respiration per unit ARL of axial roots by 50% and reduced that of lateral roots by 46% (Fig. 5). Root respiration of lateral roots per unit ARL indicated no significant difference between FL and MS lines in both high-N and low-N conditions (1.87 and 1.83 nmol CO_2_ cm^–1^ s^–1^ of FL and MS lines in high N; 1.72 and 2.10 nmol CO_2_ cm^–1^ s^–1^ of FL and MS lines in low N). Under low N, root respiration of lateral roots per unit ARL of FL lines was 67% less than that of MS lines. Lateral branching was positively correlated with respiration of lateral roots per unit ARL (Fig. 6B).

**Fig. 7.**
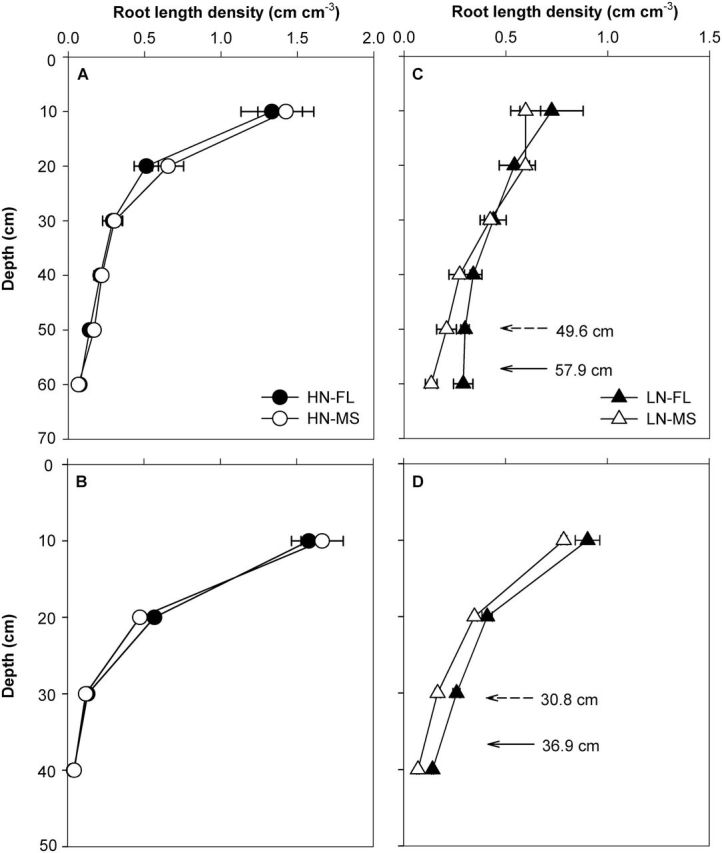
Root length density of maize lines at anthesis in the field in RS (A, C) and SA (B, D) under high N (HN, circle) and low N (LN, triangle) for five FL (filled symbols) and five MS (open symbols) genotypes. The data shown are means of four replicates of the five genotypes in each phenotype in either HN or LN ± SE. The value of D_95_ for five FL (solid arrow) and 5 MS (dashed arrow) genotypes are shown in the LN panel (C and D).

**Fig. 8.**
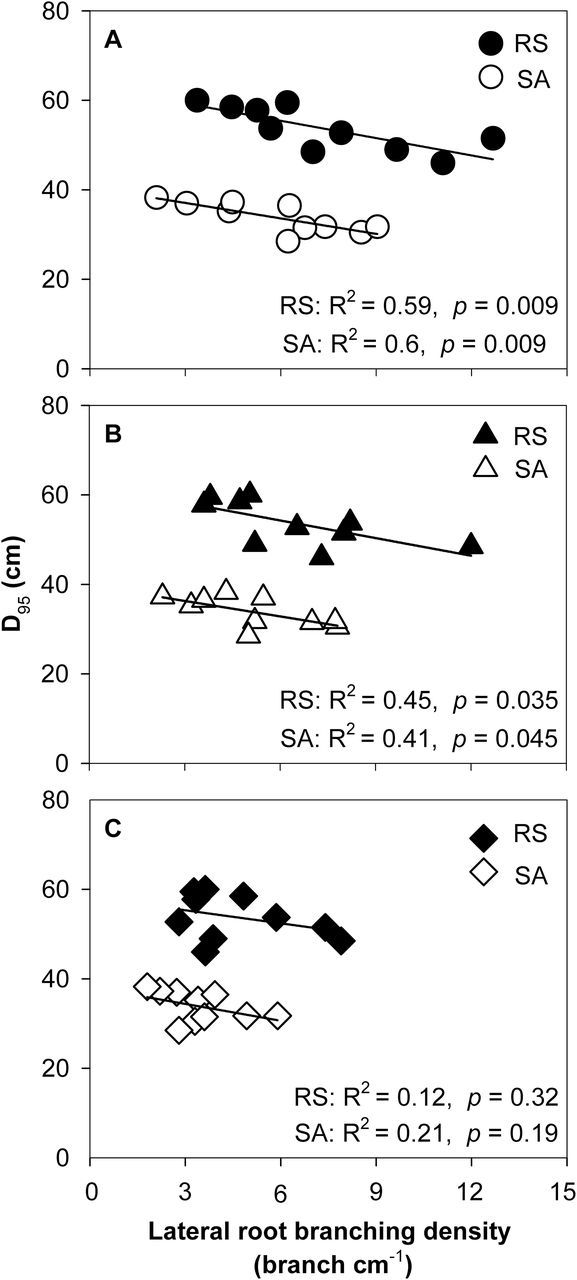
Correlation of rooting depth (D_95_) and lateral root-branching density in crown roots (A), primary roots (B), and seminal roots (C) in the field in RS and SA at anthesis. Each point is the mean of four replicates of each genotype.

Low N decreased root length density in both RS and SA (Fig. 7A–D). Under low-N conditions, FL lines had greater root length density in deep soil layers than MS lines (Fig. 7C, D). This result is also evident from the D_95_ data. Under low N in the field, FL lines had significantly greater D_95_ than MS lines (Fig. 7C-D). FL lines had a D_95_ value of 57.9cm in RS and 36.9cm in SA compared to 49.6cm and 30.8cm for MS lines. Negative relationships between D_95_ and lateral root-branching density in crown roots, primary roots, and seminal roots were found in both RS and SA (Fig. 8A–C).

### Root and shoot growth

**Fig. 9.**
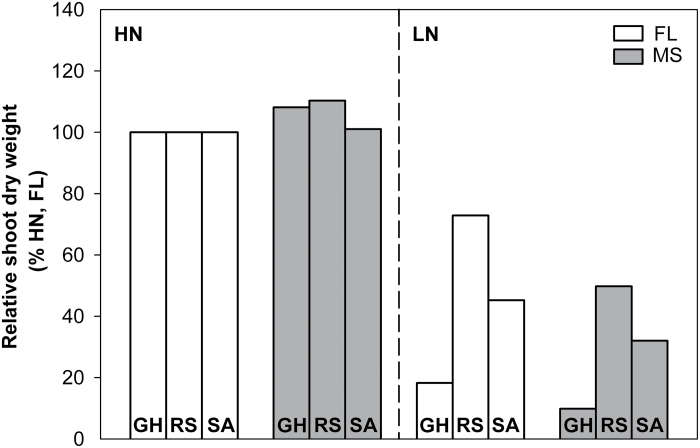
Relative shoot dry weight (% of shoot dry weight in high N) under high-N and low-N conditions at 42 days after planting in GH mesocosms in the field in RS and SA at anthesis.

**Fig. 10.**
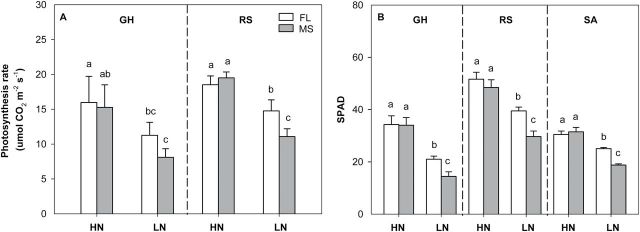
Photosynthesis rate (A) and SPAD (B) readings under high-N (HN) and low-N (LN) conditions at 42 days after planting in GH mesocosms and at anthesis in the field in RS and SA. The data shown are means of four replicates of the nine (GH) and five (RS and SA) genotypes in each phenotype in either HN or LN ± SE. Different letters represent significant differences (*P* < 0.05) compared within each location.

**Fig. 11.**
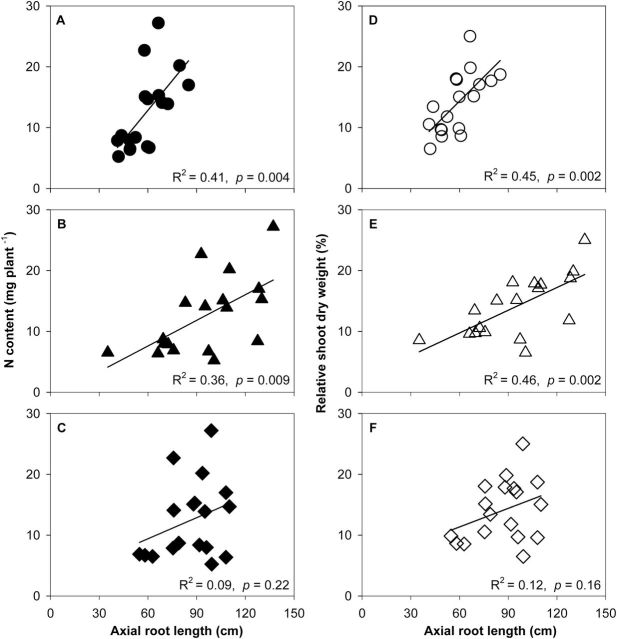
Correlation of N content (A–C) and relative shoot dry weight (% of shoot dry weight in high N) (D-F) with axial root length of the crown root (A, D), primary root (B, E), and seminal root (C, F) in GH mesocosms at 42 days after planting. Each point is the means of four replicates of each genotype.

**Fig. 12.**
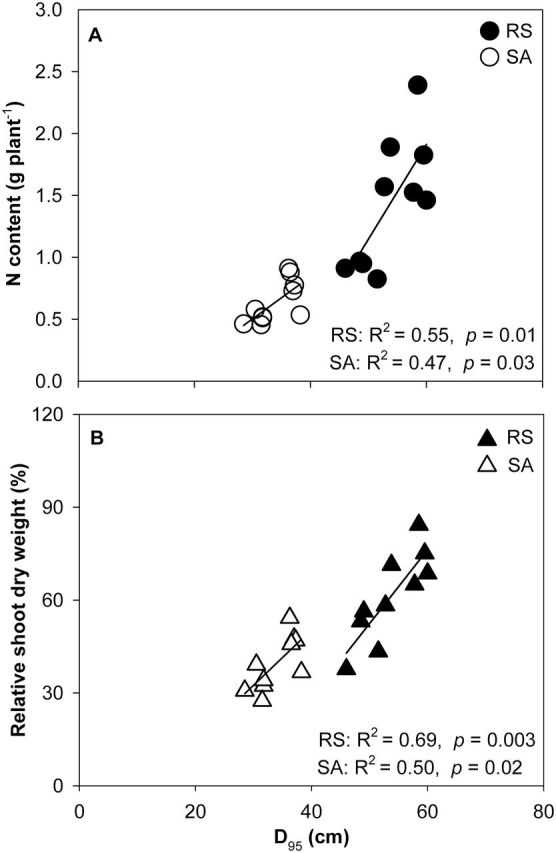
Correlation of N content (A) and relative shoot dry weight (% of shoot dry weight in high N) (B) with rooting depth (D_95_) in the field in RS and SA at anthesis. Each point is the means of four replicates of each genotype.

**Fig. 13.**
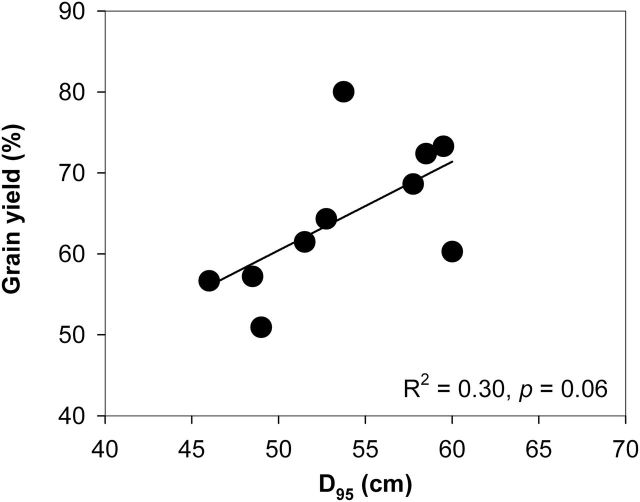
Correlation of grain yield (% of grain yield under high N) with D_95_ in the field in RS at anthesis. Each point is the mean of four replicates of each genotype.

N deficiency significantly decreased relative shoot dry weight (RSDW) compared with high-N plants in GH, RS, and SA (Fig. 9, Table 1). Under low N, FL lines had 75% more RSDW at 42 DAP in the GH, 46% more RSDW at flowering in RS, and 41% more RSDW at flowering in SA compared with MS lines (Fig. 9, Table 1). Low N reduced Pn by 38% in mesocosms and 32% in the field in the USA (Fig. 10 A, Table 1). Under low N, compared with the MS lines, FL lines had 39% greater Pn in mesocosms and 33% greater Pn in the field in the USA. Under low N, FL lines had 45, 33, and 34% greater SPAD readings than MS lines in GH, RS, and SA, respectively (Fig. 10 B, Table 1). In the mesocosms, RSDW and N content were positively related with the length of crown root and primary root axes (Fig. 11A, B), while in RS and SA, RSDW and N content were positively related with D_95_ (Fig. 12). In the field in the RS, D_95_ was positively correlated with grain yield (Fig. 13).

**Table 1.** Summary of analysis of variance for physiological parameters as influenced by N treatment and phenotype

Effect	Pn	SPAD	SNC	SDW	Y
GH					
N	147.93***	535.35***	386.38***	492.81***	–
P	1.87*	3.81***	3.26***	5.68***	–
N × P	1.43^†^	2.98***	3.36***	5.84***	–
RS					
N	90.04***	314.85***	182.04***	199.25***	98.92***
P	1.68^†^	10.36***	1.56 ^†^	3.44**	4.44***
N×P	2.64*	2.95**	3.25**	9.60***	2.59*
SA					
N	–	335.68***	386.38***	511.46***	–
P	–	5.04***	3.26***	3.70***	–
N×P	–	7.51***	3.36***	3.01**	–

Results are shown for the GH, RS, and SA experiments. SNC, shoot N content; SDW, shoot dry weight; Y, yield; N, N treatment; P, phenotype. Associated F-values and probabilities are shown (^†^, *P* ≤ 0.1; *, *P* < 0.05; **, *P* < 0.001; ***, *P* < 0.0001).

## Discussion

Our results support the hypothesis that the phenotype of FL lateral roots is superior to MS lateral roots under N limitation, as evidenced by decreased root respiration, greater rooting depth (ARL and D_95_), increased N uptake, greater photosynthesis (Pn), leaf greenness (SPAD), plant biomass, and reproductive output (Figs 3, 5, 7, 9–13). Under low N in mesocosms, FL lines had less root respiration, and greater rooting depth and shoot biomass than MS lines (Figs 3, 5 and 9). Under low N in field environments in the USA and SA, FL lines had greater rooting depth (D_95_), greater biomass accumulation and N content, and in the USA had substantially greater grain yield than MS lines (Figs 7, 9, 11–13).

For the analysis of the physiological function of lateral root branching and length, we used RILs, which are valuable in the analysis of phenotypic traits governed by multiple genes, such as lateral root branching in maize (Zhu and Lynch, 2004; Zhu *et al.*, 2005). The use of RILs ensured that the phenotypes we compared shared a common genetic background (i.e. descending from the same two parents), without artificially induced mutations or transformation events (Zhu and Lynch, 2004; Zhu *et al.*, 2005). Each RIL is a distinct genotype, and comparison of several RILs permits the analysis of a phenotype in distinct genomes, thereby reducing the risk of confounding effects from pleiotropy, epistasis, gene linkage, or other genetic interactions (Zhu and Lynch, 2004). The use of isogenic lines is not suited to the physiological analysis of phenotypic traits controlled by many genetic loci in unknown ways. In the present study, we combined results from three distinct environments, greenhouse mesocosms, and two field environments, which is noteworthy, since the field includes many environmental factors including soil biota, soil temperature gradients, soil physical constraints to root elongation, and other environmental variables that may affect results, whereas greenhouse mesocosms are more simplified environments allowing more detailed measurements. The fact that our results from RILs in greenhouse mesocosms and two field environments are all in agreement suggests that potentially confounding factors of any given environment are not driving the results.

Root systems of plants vary greatly in their architecture, from unbranched (found in many *Liliaceae* and *Orchidaceae*) to highly complex branching patterns (Fitter, 1996; Fitter *et al.*, 2002). In maize, Trachsel *et al.* (2011) observed significant genotypic variation in the density of lateral root branching, ranging from no lateral roots to 20 roots cm^–1^. In the present study, significant genotypic variation in maize lateral root branching was also found in greenhouse mesocosms and two field sites, with MS lines having significantly greater lateral root number and less lateral root length than FL lines (Figs 1 and 2). Several studies have pointed out the existence of mechanisms that control lateral root emergence and elongation. Zhang and Hasenstein (1999) reported that lateral root initiation and elongation in *Lactuca sativa* resulted from a balance between the basipetal flux of a cytokinin-like inhibitor derived from the root apex and the acropetal transport of a shoot-derived auxin that promotes lateral root development. Two pathways of NO_3_^–^ regulation of root branching were identified, one stimulating root elongation, called the localized stimulatory effect, in which the external NO_3_^–^ ion acts as a signal rather than a nutrient; and the other inhibiting branching initiation, called the systemic inhibitory effect, which appears to be positively correlated with plant tissue N level and is assumed to involve a phloem-mediated signal from the shoot (Zhang *et al.*, 1999; Zhang and Forde, 2000).

Following the economic paradigm of plant resource allocation (Bloom *et al.*, 1985), root construction and maintenance requires metabolic investment. The more roots that are initiated, the more carbon and other resources that need to be invested in root growth and maintenance, which may impair the growth of shoots and other roots, and may limit reproduction (Lynch, 2007, 2014). In greenhouse mesocosms, MS lines had greater root respiration of axial and lateral roots per unit ARL than FL lines under low-N conditions (Fig. 5), and lateral root branching was positively related with axial and lateral root respiration (Fig. 6). These results are in agreement with previous studies in maize which reported that the enhanced proliferation of lateral branches was accompanied by increased respiration of axial roots (Granato and Raper, 1989). Reduced respiration of lateral roots in FL lines would allow more metabolic resources to be available for the elongation of axial roots. Indeed, we found that FL lines had deeper crown, primary, and seminal roots in greenhouse mesocosms (Fig. 3), and a negative relationship between lateral root branching and rooting depth in greenhouse mesocosms (Fig. 4). Similar results were obtained at the two field sites. Under low N, FL lines had deeper rooting than MS lines (Fig. 7), and rooting depth was negatively related with lateral root branching at both field sites (Fig. 8). This has practical implications, since N applied early in the season as nitrate or as N forms that rapidly convert to nitrate are subject to leaching with precipitation. The rate of nitrate leaching can exceed the development of root growth in deep soil strata, which is a significant cause of low recovery of N fertilizer (Wiesler and Horst, 1993; Raun and Johnson, 1999; Cassman *et al.*, 2002; Chen *et al.*, 2011). Under such conditions, plants with deeper roots may be more adaptive by increasing N uptake from deep soil strata, thereby improving plant growth and decreasing nitrate leaching. Furthermore, greater lateral root branching places roots closer together, which increases competition for internal resources, and reduces N uptake per unit root length, as modelled by Postma *et al.* (2014).

Increased lateral root branching increased the total sink strength of the root system, but decreased the average growth rate of individual lateral roots due to internal resource limitations (Postma *et al.*, 2014). This tradeoff between the number and average length of lateral roots is also evident in a large experimental data set by Pagès and Pellerin (1994). Greater lateral root branching might increase the rate at which a soil domain is depleted, and favour the uptake of immobile nutrients like P, since most P uptake by roots occurs <1mm from the surface of a root (Nye and Tinker, 1977). This means that lateral roots spaced >2mm apart leave gaps of unexploited soil. In contrast, nitrate diffusion in soil is three or four orders of magnitude faster than phosphate. Roots 10mm apart will probably compete for NO_3^–^_ after *~*1 day (Nye and Tinker, 1977). Therefore, FL laterals capable of exploring larger volumes of soil with greater spatial dispersion among roots are better able to capture mobile resources such as nitrate. The tradeoff between lateral root branching and length suggests that plants may have a plasticity response to nitrate availability which would result in FL laterals on low-nitrate soils. These predicted plasticity responses correspond with observations also found in the modelling results by Postma *et al.* (2014), and in the literature from artificial systems (López-Bucio *et al.*, 2003; Gruber *et al.*, 2013).

Although this study focused on maize, we suggest that the phenotype of FL lateral roots would improve N capture in other species. The root system architecture of sorghum is similar to that of maize (Lynch, 2013), so the FL concept may be fully applicable. Other graminaceous species like wheat, rice, barley, and oats have the same basic root structure as maize and may also benefit from this phenotype, although greater density of nodal roots in tillering species may change the relationship of lateral root-branching density and resource capture. Our results are entirely supportive of inclusion of reduced lateral root number as a component of the SCD ideotype (Lynch, 2013). The SCD ideotype applies to both water and N capture, since both of these soil resources are often localized in deep soil strata under limiting conditions. The utility of FL phenotypes for water capture under water stress deserves investigation.

Genotypic differences in lateral root number and length have been reported in maize (Zhu *et al.*, 2005; Trachsel *et al.*, 2011; Lynch, 2013; Postma *et al.*, 2014), as we also report in this study. Previous studies indicated that lateral branching is a heritable trait (Zhu *et al.*, 2005) and genes affecting lateral branching have been identified in several species, including maize (Doebley *et al.*, 1995) and rice (Takeda *et al.*, 2003), making lateral branching and length a feasible target for plant breeding. Our results are entirely consistent with the hypothesis that FL lateral root branching reduces root metabolic costs and increases rooting depth, leading to greater N acquisition from low-N soil. Therefore we suggest that lateral root number and length deserves consideration as a trait to improve the N efficiency of maize in breeding programmes.

## Supplementary material

Supplementary Table S1. List of 18 RILs selected from the IBM population.

Supplementary Figure S1. Phenotypic variation in lateral root branching density and length under low-N conditions from the SA experimental site.

## Funding

This research was supported by the Howard G. Buffett Foundation and Agriculture and Food Research Initiative competitive grant number 2014-67013-2157 of the USDA National Institute of Food and Agriculture to JPL.

## Supplementary Material

Supplementary Data
