# Supplementary material for: Reduced frequency of lateral root branching improves N capture from low-N soils in maize
Source: J Exp Bot. 2015 Feb 13;66(7):2055–65. doi: 10.1093/jxb/erv007 (PMC4378636; doi:10.1093/jxb/erv007)
Supplement: Supplementary Data [file supp_66_7_2055__index.html]

Reduced frequency of lateral root branching improves N capture from low-N soils in maize — Reduced frequency of lateral root branching improves N capture from low-N soils in maize — Supplementary Data 

# Reduced frequency of lateral root branching improves N capture from low-N soils in maize

## Supplementary Data

Data files

**Files in this Data Supplement:**

- Supplementary Data - Supplementary Data
