# Supplementary material for: Reduced frequency of lateral root branching improves N capture from low-N soils in maize
Source: J Exp Bot. 2015 Feb 13;66(7):2055–65. doi: 10.1093/jxb/erv007 (PMC4378636; doi:10.1093/jxb/erv007)

Reduced frequency of lateral root branching improves N capture from low N soils in maize. *Ai Zhan and Jonathan P Lynch*

Supplementary Table S1. List of 18 recombinant inbred lines (RILs) selected from the intermated B73 × Mo17 population (IBM). Column displays the population of origin and RIL number for each line, while initial classification at selection based on lateral root number and length is shown in the column entitled Classification at selection. The click (√) and dash (-) means genotypes used and not used in greenhouse mesocosms (GH), Rock Spring in USA (RS) and South Africa (SA).

**Genotype**	**Classification at selection**	**GH**	**RS**	**SA**
M0067	FL	√	√	√
M0075	FL	√	-	-
M0079	FL	√	√	√
M0086	FL	√	√	√
M0096	FL	√	-	-
M0098	MS	√	-	√
M00134	MS	√	√	-
M00157	FL	√	-	√
M00182	MS	√	√	-
M00206	MS	√	√	-
M00283	MS	√	-	-
M00295	MS	√	-	√
M00303	MS	√	-	√
M00304	FL	√	√	√
M00321	MS	√	√	√
M00327	FL	√	√	√
M00361	FL	√	-	-
M00362	MS	√	√	-

Supplementary Fig. S1 Images showing phenotypic variation in lateral root branching density and length under low nitrogen conditions selected from South Africa experiment site (SA). The left panel was the many but short (MS) phenotype (M0321, A-F), and right panel was the few but long (FL) phenotype (M0304, G-L). In each panel, images from up to down were the 5th, 4th, 3rd, 2nd, 1st whorl of crown root, and the primary and seminal root, separately. The representative roots of MS and FL were selected from the same replicate.

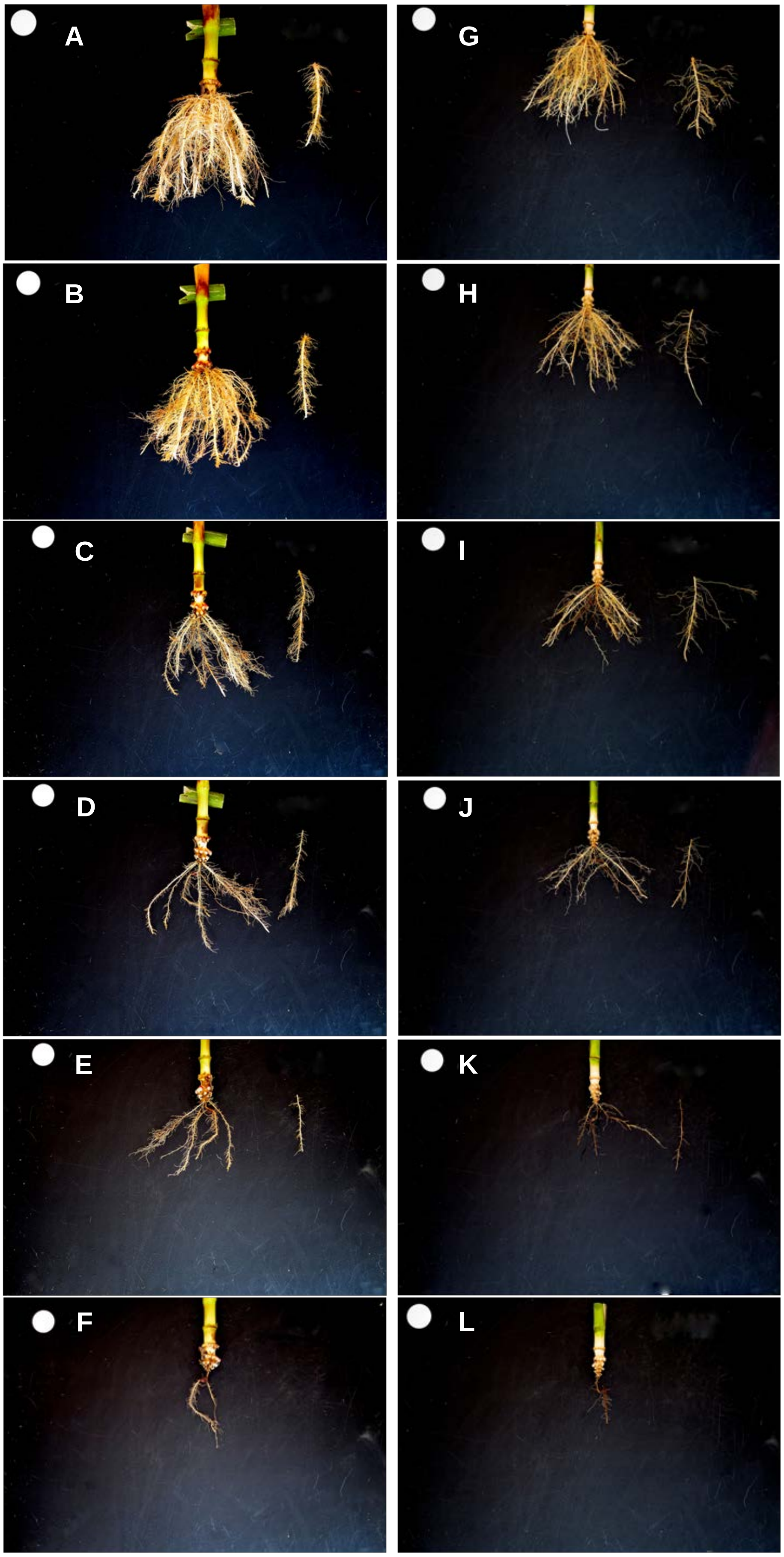

Supplement: Supplementary Data [file supp_erv007_jexbot136499_file001.pdf]
